# Acceptability and Feasibility of Sharing a Soapy Water System for Handwashing in a Low-Income Urban Community in Dhaka, Bangladesh: A Qualitative Study

**DOI:** 10.4269/ajtmh.17-0672

**Published:** 2018-06-11

**Authors:** Farhana Sultana, Leanne E. Unicomb, Fosiul A. Nizame, Notan Chandra Dutta, Pavani K. Ram, Stephen P. Luby, Peter J. Winch

**Affiliations:** 1 International Centre for Diarrhoeal Disease Research, Bangladesh (icddr,b), Dhaka, Bangladesh;; 2 University at Buffalo, Buffalo, New York;; 3 Stanford University, Stanford, California;; 4 Johns Hopkins Bloomberg School of Public Health, Baltimore, Maryland

## Abstract

Handwashing with soap at key times is an effective means of reducing pathogen transmission. In a low-income community in urban Dhaka, we piloted and evaluated the acceptability and feasibility of a shared handwashing intervention. This included promotion by community health promoters of a homemade solution of detergent powder mixed with water and stored in a 1.5-L reclaimed mineral water bottle. Community health promoters encouraged sharing of the recurrent detergent cost among compound members. Of 152 participating compounds, fieldworkers randomly selected 60 for qualitative assessment. Fieldworkers conducted 30 in-depth interviews and five focus group discussions among purposively selected compound members. The reclaimed bottles served as an easily accessible dispenser for the soapy water, which could feasibly be retained next to the toilet and kitchen areas for communal use. Bottles functioned as a positive reminder for handwashing at recommended key times. Most compounds (45/60, 75%) shared a common soapy water system and its associated costs. There was reluctance to prepare soapy water for shared use in the remaining 25%. Soapy water was an acceptable hand cleaning agent, with the bottle as a feasible dispenser. It was simple in design, cost-effective, replicable, popular with intervention recipient, and neighboring nonrecipients, and commonly shared among nonrelated households. The need to share expenses and product preparation served as a barrier. Developing a sustainable maintenance system, therefore, is critical to ensuring the public health benefits of handwashing with soap.

## INTRODUCTION

Diarrhea and respiratory infections are common causes of morbidity and leading causes of death among children aged less than 5 years, particularly in low- and middle-income countries.^1^ Handwashing with soap can reduce diarrhea and acute respiratory infections among children aged less than 5 years.^2–4^

Studies conducted in Bangladesh demonstrated that handwashing with soap significantly reduces childhood diarrhea.^5^ However, handwashing is infrequent in low-income countries,^6,7^ including Bangladesh,^8,9^ in part because of the high cost of cleansing agents relative to household income.^10–17^ Additional barriers to maintaining bar soap at a handwashing station include fear of theft and reluctance to share costs and maintenance duties among households using a common water source and sanitation facility.^12,15^

Soapy water is an alternative to bar soap previously promoted in Kenya, Peru, and Bangladesh.^18–20^ Soapy water is produced by mixing detergent or other soap with water in bottles. The resultant solution costs less than bar soap, is an easy-to-make, and convenient hand cleaning agent.^19–21^

Intervention trials in Bangladesh identified soapy water as an acceptable and feasible option of soap formulation,^17^ with 60–70% uptake both in rural and urban communities.^18,22^ Formative research indicated that low-income urban community members in Dhaka were not habituated to wash hands using soap and water, and this could be addressed through handwashing intervention that included promotion of soapy water for handwashing.^17^ However, we lack data on feasibility, convenience, and sustained use of sharing soapy water bottles in these low-income urban households where shared handwashing sites are the norm. This article examines the acceptability and feasibility of sharing the soapy water system as a cleaning agent and dispenser in a low-income neighborhood in Dhaka, Bangladesh, drawing on data from a qualitative assessment conducted during a pilot handwashing intervention,^17^ from a systematic review on sustained adoption of Water, Sanitation, and Hygiene (WASH) behaviors^23^ we conducted, and other relevant literature we reviewed on promotion of water, sanitation, and hygiene.^17,18,21,24–35^

## METHODS AND MATERIALS

We introduced the handwashing intervention in September 2010 that continued until February 2012 in the low-income Mohammadpur community of urban Dhaka. The intervention consisted of three components: provision of hardware (water drum with taps so that water for handwashing is always present), promotion of soapy water system, and implementation of a behavior change strategy to promote handwashing with soap (Table 1). This article focuses on the drivers and barriers to sharing the costs and maintenance of the soapy water system.

**Table 1:** Handwashing enabling technologies evaluated in a low-income urban community in Dhaka, Bangladesh

Photo	Name (capacity)	Cost	Description
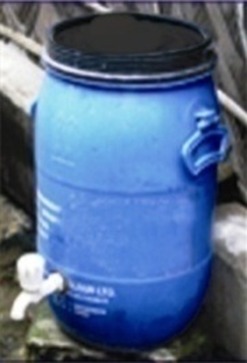	Drum (30 L)	US$6.25	The drum consists of a plastic barrel with a turn-handle water-dispensing spout that was provided to store and ease washing both hands. It provided a consistent running water supply, even during interruptions in municipal water supply, and because it was always available for handwashing, unlike other water sources which serve many purposes and were not always available for handwashing (e.g., tube wells used for laundry, bathing, and dishes).
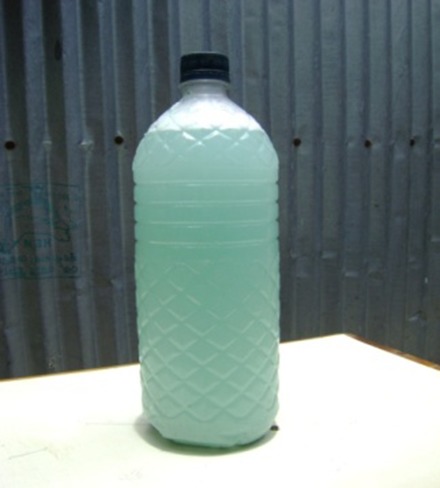	Soapy water bottle (1.5 L)	US$0.06	The soapy water bottle consists of a reclaimed 1.5-L commercial water bottle. A small hole was drilled in the top of the bottle’s original cap to dispense soapy water agent. Thirty grams of detergent powder was mixed with water to prepare soapy water liquid for handwashing. We tested this technology as it provides soap and water together as a low-cost alternative of bar soap or hand cleansing agent in a single package and produced from local materials. This combination is popular because it is easy and cheap, and there is little risk of theft, unlike the drum.

This table appears in color at www.ajtmh.org.

### Study setting and participants.

Mohammadpur is a subdistrict (*thana*) of Dhaka city with a variety of housing arrangements. Three low-income neighborhoods of Mohammadpur, Shyamoli, Sunibir-1, and Sunibir-2 with approximately 25,000 residents were included in the study. The residents inhabited household compounds, which were typically overseen by a compound manager, who was generally the landlord or a representative of the landlord.

Compounds typically included a main entrance and a long hallway with either one or opposite-sided multiple doors. Each door opened to a single room occupied by a separate household. Most households had no family ties to their neighbors; households relocated frequently. Most rooms were made of brick with a tin roof, and households shared a common courtyard, access to cooking areas, between one and three water sources located outside of the courtyard, and sanitation facilities with neighboring households. Soap for personal use was typically not kept next to the toilets but in residents’ rooms, and therefore not accessible immediately after toileting. Soap for shared use among neighboring households was usually not available. Study fieldworkers purposively selected 152 compounds consisting of 8–10 households across these three neighborhoods based on their availability and willingness to participate in the study, and all 152 compounds received the soapy water system intervention.

### Intervention design.

With the collaboration of a local non-governmental organizations named Dushtha Shasthya Kendra, we recruited seven community health promoters and one supervisor from the intervention area to lead and monitor the handwashing promotion. We invited them to the International Centre for Diarrhoeal Disease Research, Bangladesh (icddr,b) office and held a 3-day basic training on how to conduct and deliver behavior change communication sessions using flipcharts and cue cards. Training methods included both classroom and practical sessions during which the community health promoters learned and practiced the message delivery process and conducted an additional 1-day field testing of the materials and role played at Mohammadpur community. This training of community health promoters aimed to create resource person in the community to play an effective role for handwashing behavior change.

The community health promoters were responsible to conduct courtyard meetings once a month in each compound and included at least one member from each household. They also delivered interpersonal communication sessions twice a month using flipcharts and cue cards among households with a child less than 3 years old. They targeted the mother of children < 3 years old to deliver the sessions as they were expected to reach an age of 5 years after the 2-year study and intervention period.

**Figure 1.**
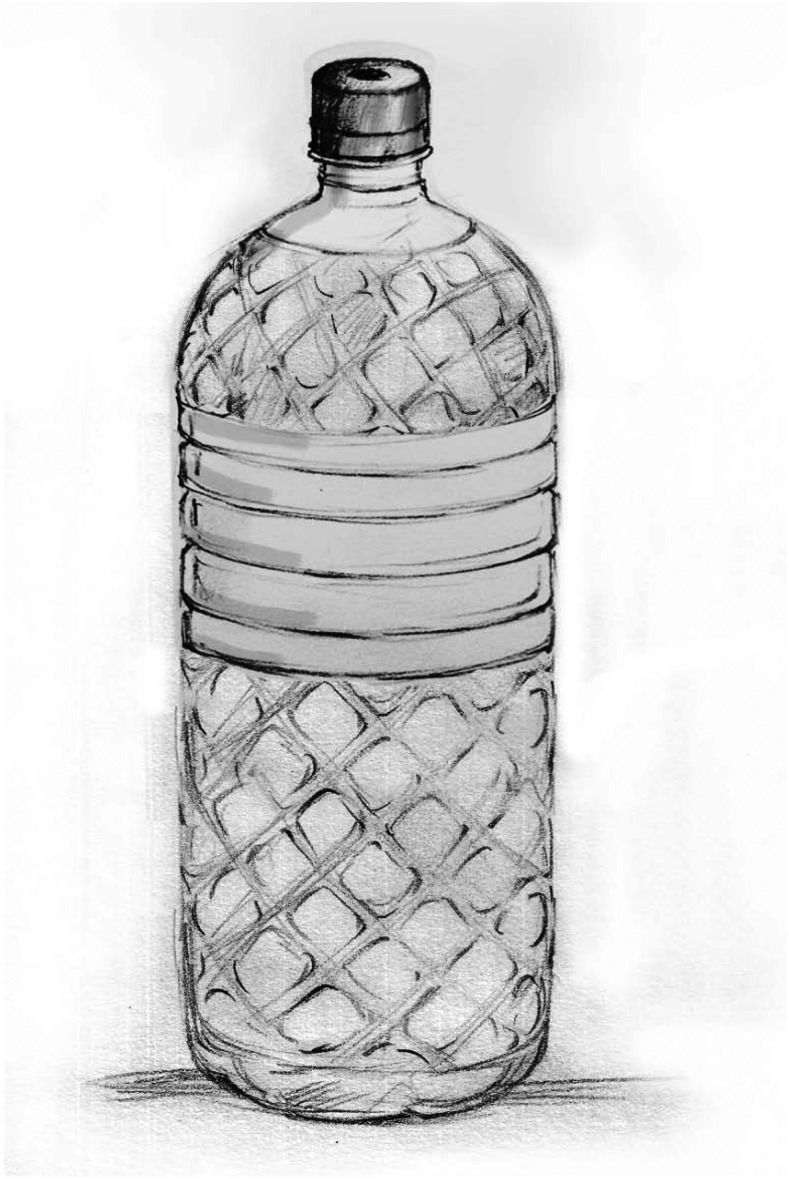
Reclaimed bottle with a small hole drilled on the top to dispense soapy water.

**Figure 2.**
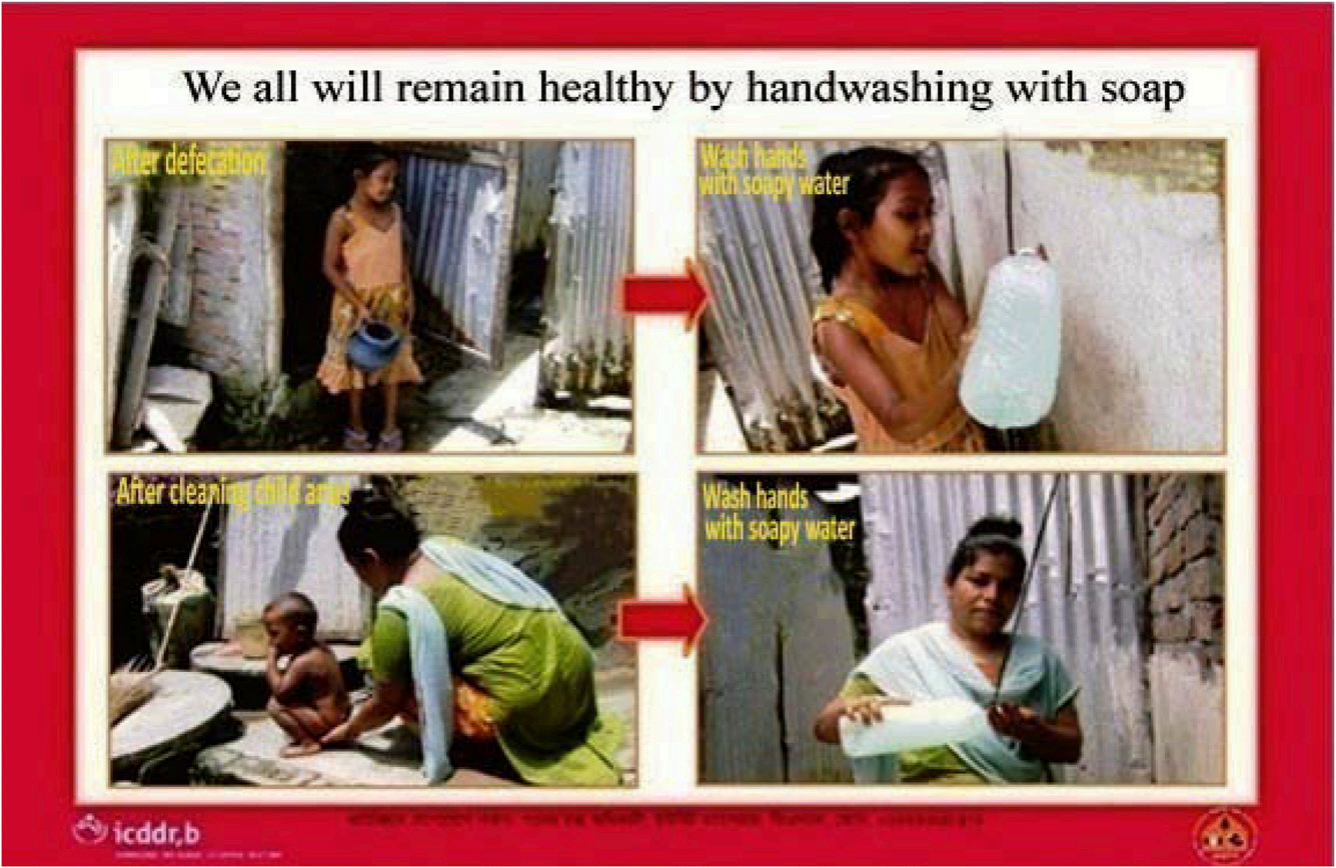
Cue card depicting health benefits to promote handwashing behaviors after defecation and after cleaning a child’s anus among low-income urban community in Dhaka, Bangladesh.

The intervention hardware design has been described previously.^17^ Briefly, the soapy water system combined two elements: 1) soapy water as handwashing agent: adding a 30-g packet of powder laundry detergent to 1.5 L of water and 2) soapy water bottle as dispenser: a 1.5 L-reclaimed bottle previously used for commercial drinking water with a small hole drilled on the top to dispense soapy water (Figure 1). We provided the dispenser to the respondents, and they assumed the cost of the handwashing agent and were encouraged to wash hands 1) after defecation and 2) after cleaning a child’s anus and/or contact with feces. We provided respondents with laminated A4 size cue cards depicting our recommended behaviors (Figure 2) that the community health promoters fixed next to the toilet and handwashing stations or water collection sources to remind the compound members about handwashing.

We encouraged shared use and maintenance of the soapy water system. Household members already shared common water, sanitation, cooking, and bathing facilities with the neighboring households. Fieldworkers proposed that the compound managers be responsible for the maintenance and preparation of the soapy water system for feasible and consistent use.

### Study design and sampling.

Fieldworkers prepared a list of all 152 enrolled compounds from the three areas and randomly selected 60 compounds (20 from each area) for spot checks to assess handwashing facilities. To do so, they administered a short questionnaire among compound members to explore whether the soapy water bottle was present and maintained for daily handwashing purposes and to identify compound members that did or did not share and use the common soapy water system.

We then conducted a qualitative assessment of attitudes and perceptions among the household members of these 60 compounds who used the soapy water system and among those who chose not to use it. Fieldworkers obtained detailed feedback about the acceptability and feasibility of the intervention for washing hands to inform refinement to the system and its promotion. We purposively selected a total of 70 respondents and included at least one from each of 60 compounds based on their availability at home at the time of data collection, and willingness to participate in the study interviews for this qualitative assessment.

### Data collection.

We structured the study questionnaires (Table 2) using the Integrated Behavioral Model (IBM) for WASH that theorizes the adoption of new WASH behavior and technologies are influenced by the interconnected contextual, psychosocial, and technological dimensions.^36^ The contextual dimension includes social and physical environment (e.g., access to water, sanitation, and hygiene facilities) in which the WASH behaviors and technologies are implemented, the psychosocial dimension includes social and psychological factors (e.g., collective efficacy to wash hands) that affect WASH practices, and the technological dimension includes factors (e.g., physical products and use of products such as soapy water system) affecting adoption of WASH technologies. We used these dimensions to guide the interview and assess the soapy water system.

**Table 2:** Example of interview guideline of soapy water system study in a low-income urban community in Dhaka, Bangladesh

Question type	Subquestions with relevant IBM dimensions
General questions regarding handwashing practices	1. Describe your handwashing practices? Where and when do you generally wash your hands? (contextual dimension)
	2. When and how did you and your compound members wash their hands before receiving this intervention? When and how do you wash their hands now (Explore all practices during key times of handwashing: after defecation, after cleaning child anus)?(contextual dimension)
Questions relating to handwashing intervention	3. What is your opinion about this handwashing intervention? Do you think the intervention made any change to handwashing behavior? What kind of change, please describe. (technological dimension)
	4. Do you use the soapy water system? After what activities are you use it most? What aspects of the handwashing intervention you like or dislike most? (psychosocial and technological dimension)
	5. What are the advantages and disadvantages of using the soapy water system? Do you have any preference regarding the shape, design, and maintenance? (psychosocial and technological dimension)
	6. How did your neighbors react to this handwashing intervention? Were they interested? Did they want one for their own? (psychosocial dimension)
	7. Does the soapy water system help your family members? Do they like using it? (psychosocial dimension)
	8. How did you know about this intervention? Who came to visit? How did she discuss, group or individually? Which approach did you like (interpersonal or courtyard session)? (psychosocial dimension)
Questions identifying issues surrounding use of the handwashing station	9. What are the topics and with what materials were discussed during the intepersonal or courtyard sessions? What do you think about the role of the CHPs? (psychosocial dimension)
	10. Who takes the responsibility from the compound for optimum use of this intervention? Who refills the soapy water and drum regularly? Did you face any problem regarding maintenance (refilling and others…) these handwashing stations? If yes, how did you solve it? (psychosocial dimension)
	11. What are the perceived factors/constrains that prevent optimal use of this intervention? (psychosocial dimension)
	12. What are the impacts of the intervention on health of the compound members, especially on children? (*Probe for both negative and positive impact the respondent wants to mention*.) (psychosocial dimension)
	13. What do you think about the sharing of soapy water system in a compound? Is it possible? Why or why not? (technological dimension)
	14. What was the attitude of those who do not use the soapy water system? What do you suggest as a compound manager to involve them in this intervention? Do you have any role regarding this? (technological dimension)
	15. Can you play any role as a compound manager to continue the handwashing practices inside or outside your compound? What role? (contextual and technological dimension)

IBM = integrated behavioral model.

Eight experienced and trained qualitative researchers collected data between August 2011 and February 2012. They conducted the spot checks of facilities in August–September 2011, and the in-depth interviews and focus group discussions in January–February 2012. Based on results from spot checks of facilities, they collected data until data saturation was reached and conducted 30 in-depth interviews: 15 with the shared users, eight with the nonusers, and seven with the compound managers. They also conducted five focus group discussions with 40 respondents (eight in each discussion): two with the shared users, two with the nonusers, and one with a compound managers. Qualitative researchers investigated perceptions of the strengths and weaknesses, and the acceptability and feasibility of sharing a soapy water system throughout the 18-month intervention period.

The in-depth interviews averaged 45 minutes and focus group discussions averaged 60 minutes. We conducted all the interviews in Bengali at the respondents’ homes at their preferred time and digitally recorded them to avoid missing information. Soon after the interview was conducted, we prepared verbatim Bengali transcripts and fully translated them into English.

### Data analysis.

We drew on inductive (emergent) coding techniques from grounded theory, combined with a priori (predefined) codes based on the IBM-WASH framework to guide our qualitative data analysis. We created a priori codes representing the contextual, psychosocial, and technological dimensions in the IBM-WASH framework shown in Table 3) and then developed code tree of emergent codes (such as shared use of soapy water system, soapy water as hand cleansing agent, and the bottle as a dispenser as described in the results section).

**Table 3:** Conceptual framework of soapy water system for handwashing promotion in a low-income urban community in Dhaka, Bangladesh: based on the qualitative findings analyzed using the Integrated Behavioral Model for Water, Sanitation, and Hygiene

	Contextual	Psychosocial	Technology
Societal/structural	Existent national policy promotes handwashing with soap. However, water supplied by the Water and Sanitation Authority at Dhaka municipality was irregular.	Compound managers willingly wanted to lead the maintenance and preparation of soapy water system that created ownership of the intervention.	Soapy water system did not require manufacturing, financing, and distribution as reclaimed bottles used as a technology.
Community	Frequent access to market and resources. Water, sanitation, and cooking facilities are shared among compound households.	Lack of social integration among compound members caused discord taking responsibility in preparing and maintaining the soapy water system for communal use.	Some respondents preferred individual use of soapy water system because of the lack of communal maintenance.
Interpersonal/household	Management of compound and shared water and sanitation facilities was the compound managers’ responsibility.	Neighborhood compound members that did not receive the intervention prepared their own soapy water system for handwashing.	Sharing of access to product was successful among most of the compound members that paid for and prepared soapy water for shared use.
	Water management, cooking, cleaning, and child nurturing are women’s roles in the household level.	Mothers teach their child to wash hands.	
Individual	Men are primary earners and women take care of household works.	Study participants had self-efficacy to use the soapy water system and handwashing with soapy water. They were disgusted sharing soapy water bottle with other compound members for handwashing, especially after defecation.	Detergent to prepare soapy water required low cost compared with bar soap and feasible to store in convenient places such as next to the kitchen and toilets.
	Younger age affected the use: played with the soapy water bottle, dirtied, emptied, and destroyed them frequently.		
Behavioral/habitual	Enabling habit formation of handwashing with soapy water system.	Existing handwashing habit with laundry soaps replaced by soapy water system. Study participants expected health benefits outcome to reduce diarrhea.	Time saving, no risk of theft, and easily accessible for both child and adult. Soapy water system was also effective for routine use and created supportive environment as the product itself worked as a reminder handwashing.

After coding, we summarized and analyzed the overall themes collectively to draw inferences. We also performed thematic content analysis to further characterize our results. First, we analyzed each in-depth interview and each group discussion separately. Subsequently, we compared and cross-checked them with the shared versus nonusers and with compound managers for consistency. The combination of predefined codes representing the three dimensions in IBM-WASH, with inductive thematic coding of interview transcripts, served to contextualize the feasibility and acceptability of soapy water system. We did not attempt to quantify the results because small numbers of respondents were interviewed.

The predefined codes for 1) the contextual dimension in IBM-WASH include physical characteristics of the Mohammadpur community, 2) the psychosocial dimension includes respondents’ perceptions and behaviors toward handwashing using the soapy water system, and 3) the technological dimension includes the soapy water system itself (Table 3).

### Ethics.

Fieldworkers obtained informed written consent from the respondents before data collection, and this protocol was reviewed and approved by the icddr,b Ethical Review Committee.

## RESULTS

The majority (54/70) of our in-depth interview respondents were female as the data collection occurred during day time when most male members were out for work. Their median age was 27 years; one quarter of them had not attended school. Most respondents were homemakers and others were involved in small business and paid employment (Table 4). In the analysis of 30 in-depth interviews and five focus group discussions, we found similar responses and behaviors related to the use of the soapy water system for handwashing behavior change and have summarized these together. The themes emerging from the inductive coding inform our understanding of the strengths and weaknesses of sharing the use, costs, and maintenance of the soapy water system. We now present data on utility of the bottle as a soap dispenser and cue to action, soapy water as an affordable and convenient hand cleansing agent, and finally sharing and perceived barriers of sharing a soapy water system.

**Table 4:** Sociodemographic characteristics of the respondents of the in-depth interview of the soapy water system study in a low-income urban community in Dhaka, Bangladesh

Sociodemographic	Total *N* = 70	
	*N*	(%)
Gender of the respondent		
Female	54	77
Type of the respondent		
Compound members	55	79
Compound manager	15	21
Age of the respondent		
19–35 year	38	54
Above 36 year	32	46
Education of the respondent		
No education	25	36
Primary	21	30
Secondary	17	24
Above secondary	7	10
Occupation of the respondent		
Housewife	54	77
School teacher	1	1
Business	12	17
Service	3	4

### Convenience of soapy water “bottle” as a dispenser for shared use.

During the in-depth interviews and focus group discussions, the most commonly stated strength of the soapy water bottle was its availability as a dispenser which facilitated shared use. The bottles were reported as easily accessible and user-friendly devices that facilitated sharing the bottle for handwashing among all the respondents who participated in the study. Therefore, they kept this device at a convenient location where it could potentially be shared by other compound members and reported that similar reclaimed bottles were easily obtainable if they wished to make additional dispensers themselves.

Retaining soapy water bottles at multiple places, such as next to the toilet, near water sources, and in the kitchen, was considered more conductive for sharing than placing bar soap there. Most respondents reported that they did not have a specific place to keep individual soap bars in the shared compounds and bar soap was likely to be stolen by other compound members, crows or rats. Therefore, they kept bar soap inside their houses, which they perceived as a safe place and after each use they would return the bar soap back to that place, which they found inconvenient and a nuisance that resulted washing their hands with only water. One female respondent during the in-depth interview said: “*We benefit from the soapy water bottle, because the cost of both detergent and the reclaimed bottle are very low and thus have no risk of theft like bar soap while kept outside the room. It is also worth using because crows cannot take the soapy water bottle away as they do with bar soap. So, we are not worried keeping the soapy water bottle outside.”*

Most respondents reported that the introduction of the soapy water “bottle” also worked as a reminder to wash hands at recommended key times, especially after defecation, that enhanced sharing and use among residents of all ages. Retaining the bottles next to the toilet and cooking or courtyard areas worked as behavioral stimulus to wash hands; bar soap was rarely placed in a shared location. Some respondents reported that children < 5 years old were enthusiastic to wash their hands using soapy water, and they reminded their mothers to wash hands at recommended key times. One female respondent during the focus group discussion said: *“Since soapy water bottles were introduced for handwashing, we remember the key times while seeing it around the compound. The bottle made handwashing a part of our daily life.”*

Another female respondent during the focus group discussion said the following: “*None kept soap next to the toilet or water points before soapy water bottles were introduced. At that time, we used to return inside the room to get bars of soap, thus we were unmotivated, and handwashing was rather difficult compared to now. Now we can keep the soapy water bottle beside the water point or inside the toilet instead using bar soaps.*”

### “Soapy water” as a low-cost hand cleansing “agent” for shared use.

Most of the respondents that participated both in the in-depth interviews and focus group discussions found the 30-g detergent used for making the soapy water agent to be affordable and allowed sharing the cost among compound members. It cost $0.025 for a 1.5-L bottle of soapy water which the participants perceived lasted longer than bar soap as they used one bottle of soapy water ≈40 times compared with a 100 g bar of soap that costs $0.35. Respondents often stated that they reserved the soapy water for handwashing and used costlier bar soap or laundry bar for bathing. Some respondents also reported they were using two packets of detergent powder (60 g for a 1.5-L bottle of soapy water) for greater foam generation of the soapy water as a hand cleansing agent. Most respondents reported low risk of contamination or misuse during the frequent use when shared by other members, compared with bar soap. They added that they did not touch the soapy water agent itself. They stated that the bottle protected the soapy water agent inside, allowing them to avoid direct contact with the hand cleansing agent. This was not possible when using bar soap. One female respondent during the focus group discussion said: *“I feel disgust to use the same soap that is used by many people after defecation because the soap becomes contaminated. However, the soapy water bottle does not have such problem, because people touch the bottle not the soapy water inside. We feel soapy water is a better option compared to bar soap.”*

During focus group discussion, some respondents reported that nearby compounds who did not receive the intervention liked the idea of preparing soapy water and adopted the soapy water system for handwashing. Because it was easy to make, and ingredients were readily available, the female members of those compounds prepared their own soapy water systems for individual family use.

### Sharing a soapy water system.

During spot checks, the soapy water system was present, and bottles were filled with soapy water among 75% (45/60) of the randomly selected compounds. During both in-depth interviews and focus group discussions, these compound members who shared a common soapy water system with their neighboring households mentioned that it created a supportive handwashing environment as it provided an innovative alternative to bar soap and a new and easy homemade solution for handwashing, and included intensive encouragement by the community health promoters, which were effective for the routine use of the system. One compound manager during the in-depth interview said: *“The compound members are sharing soapy water bottle as it made our handwashing practices easier. We (households) rotate covering the cost, preparation (soapy water) and cleaning (soapy water bottle) responsibilities as it is convenient.”*

### Role of the managers for shared use of soapy water system.

The role of compound managers who led and allocated the preparation and maintenance of the soapy water system was an important factor that encouraged shared use of the soapy water system. One female respondent during the in-depth interview said: *“The compound managers are mainly responsible to maintain the soapy water system as it is a common device for our shared use. They prepare soapy water, refill and clean the bottle. Nowadays, even children and the male members of our compound got encouraged to maintain the soapy water system.”*

### Perceived barriers to sharing soapy water system.

There were 25% (15/60) of the randomly selected compounds where members did not share the system and commonly reported about some barriers of sharing the system.

During the focus group discussions, most of these households found preparing soapy water an additional burden over and above their other routine responsibilities, and were unwilling to prepare soapy water to share with others, or to pay for and to share responsibility for soapy water preparation. This caused discord among compound members who did not have an agreement among themselves and with the compound manager on who would pay and prepare soapy water. Rather they reported that they prepared and kept their own soapy water system in their rooms for individual use among their own household members. One female respondent during the focus group discussion said: *“I ask others for detergent to prepare soapy water for shared use. Some households provide me detergent, some do not. What to do if some do not? I pay from my own pocket, and it does not matter for me since it is only 2 taka (US$0.025). Well, such 2 taka is wasted in so many ways that we never realize, though spending for soapy water is something good according to me since if I spend 2 taka, all others can wash hands.”*

Left hands are commonly used for cleaning the anus with water after defecation in Bangladesh. Thus, some respondents that did not share the soapy water bottle also reported that compound members were touching the bottle soapy water bottle kept next to the toilet with their left hands after coming out from the toilet and thought that they may have contaminated the bottle with their feces. One female respondent during focus group discussion expanded: *“The soapy water bottle is used after defecation, and it feels disgusting to wash my hands at another time. Moreover, the bottle doesn’t look neat and clean as it is used frequently by several persons after defecation.”*

During the in-depth interviews and focus group discussions, some respondents also reported that the indentations on the surface of the available reclaimed bottles became dirty very quickly from frequent use which served a barrier for sharing. They reported that people from different households use the bottle and it became difficult to maintain and keep clean. Thus, they found poor bottle cleanliness disgusting preventing them sharing with other households, whereas some did not like the way bottles became dirty but continued to share. They also mentioned that the younger children commonly play with the empty bottles, and destroy them. Therefore, the compound needs to frequently replace the reclaimed bottles for further soapy water preparation and use. One female respondent during the focus group discussion said: *“Children use the bottle repeatedly and it becomes dirty soon. I assume changing the outer side of the bottle into plain or simple shape would be better to clean.”*

Some respondents who did not share found the size of the bottle too small for communal use or sharing among more households because many people wash hands and the soapy water was used up quickly and required frequent refilling.

## DISCUSSION

We found that three quarters (75%) of the households shared the soapy water system and covered the recurring cost of detergent (US $0.03/1.5 L), up to 18 months after initiation of the intervention, suggesting that unrelated families in urban compounds were comfortable to share the soapy water system with others in contrast to not sharing bar soap.^12,15^ Therefore, the promotion of soapy water as a hand cleansing agent with 1.5-L bottles as a dispenser was low-cost, acceptable, and feasible for handwashing in low-income urban communities, and has the potential for sustained adoption.^23^ This mirrors the findings of a study conducted in rural Bangladesh.^18^ In addition, a trial among low-income urban communities in Dhaka found soapy water was equally effective as bar soap in removing indicator organisms from hands and more effective than water alone.^35^

The soapy water system minimized the barriers of high-cost and theft associated with bar soap; though difficulty in using, stocking, and maintaining the soapy water system as a shared good was a persistent barrier for one quarter (25%) of the households. Those who did not share reported that they prepared their own soapy water system for individual household use because of the lack of communal agreement on covering the cost and preparation of soapy water for shared use.^37^ Such barriers to sharing are common in urban environments that lack space for hygienic facilities and soap in latrines,^24^ where most neighbors are not related, and moving house is frequent. This reduces the ability to build on mutual understanding, social ties and relationships to enforce new and more healthful norms.^17,38–40^ For example, shared toilets are often unclean, and filthy to use resulting in them becoming non-functional.^41,42^ However, by promoting commitment to handwashing with soap, and changing the negative attitudes to increase shared toilet users’ collective participation in cleaning and sharing was possible, which can possibly increase the shared use of soapy water system.^37^

The analysis based on the IBM WASH framework showed that the soapy water system was a technology that provided accessibility, and created a supportive environment and feelings of ownership among 75% of the compounds (Table 3). In addition, the monthly/bi-monthly sessions by the community health promoters likely supported the willingness to share the soapy water bottle. The psychosocial level factors were shared values and norms for handwashing, cooperation, and collective efficacy for compound-wide use of soapy water bottles to reduce diarrhea, and commitment to pay and prepare soapy water among the compound members and managers in maintaining and sharing the soapy water system (Table 3).^43,44^ Soapy water is one of several WASH technologies that can be shared. Table 5 summarizes findings on sharing of water sources, toilets, and soapy water of published papers. The table demonstrates that to some extent, there are common factors involved in sharing, and these include design of the technology, responsibilities for cost and maintenance, ownership, accessibility, and user satisfaction (Table 5). For shared treated water, decision on placement, design, and water supply were considered important. For shared latrines, cleaning, water supply, and disposal were barriers for shared use. Among other studies of soapy water, sharing was affected by design and preparation (Table 5). The soapy water bottle was a low-cost technology (US$0.06 that could be reused as long as it continued to hold soapy water),^35^ it was easy to make and compound members had no fear that it would be stolen or moved,^18^ so sharing was feasible, which thereby enhanced convenience (Table 5).

**Table 5:** Comparison of factors proposed to affect sustained sharing of water, sanitation, and hand hygiene facilities by unrelated households

Factors affecting sharing	Shared water points	Shared toilet	Shared soapy water
No. of studies (no. From Bangladesh)	*N* = 4 (2)	*N* = 7 (0)	*N* = 4 (3)
Decision on placement	4	1	3
Maintenance factors			
Recurring cost	2	5	4
Design/technology	3	5	4
Cleaning	N/A*	6	0
Repair	3	2	2
Preparation	N/A	N/A	3
Chlorination	3	N/A	N/A
Water supply	3	5	3
Disposal system	N/A	4	N/A
Construction	2	5	1
Other factors			
Ownership	3	5	2
Landlord/household interest	3	3	1
Acceptability and satisfaction	4	7	4
Accessibility/availability	4	6	4
Further modification	4	4	1
References	23–26	27–33	17,18,21,34

* The factors are not applicable for either water, toilets or soapy water, for example shared water points does not need disposal system and shared toilets does not need chlorination.

Developing a sustainable maintenance system along with behavior change messages fostering the shared social norms, perceived disgust, and good citizenship requires addressing issues associated with difficulty in preparing and sharing of soapy water system, especially in settings where people share water and sanitation facilities.^45^ Including landlords and compound managers as primary audience for an intervention, for example engaging them to lead sessions^46^ or oversee management of shared resources found to be an effective means for cleaning and maintain shared toilets^47^ and can be considered for soapy water system promotion in the future.

**Figure 3.**
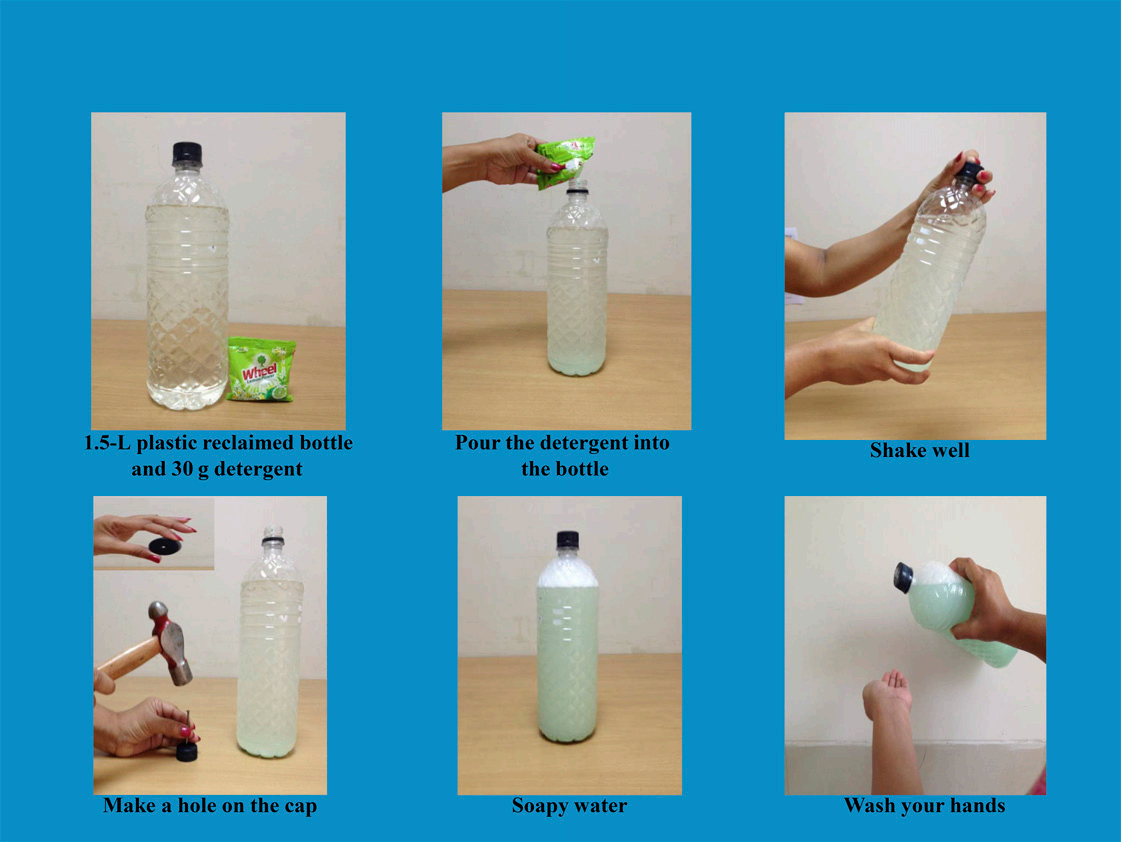
Soapy water preparation method.

Soapy water as a “hand cleansing agent” varies in terms of soap type, concentration, and preparation method.^20,35^ Soapy water has been prepared from bar soap for use in tippy taps,^48^ or alternatively from liquid soap^20^ or shampoo though cost could be a barrier for these alternatives. We instructed people to use a 30 g detergent packet to prepare 1.5 L of soapy water (Figure 3), though some respondents used two packets per 1.5 L bottle as they were concerned that insufficient foam was generated using the standard formulation, which doubled the cost ($0.05) but was still less than bar soap ($0.35). This finding is consistent with a randomized trial in urban Dhaka, Bangladesh.^35^ Although there is a lack of data on the number of handwashing occasions using bar soap, assuming that, as reported by study participants, even if one bottle of soapy water lasts for as many episodes of handwashing as a single bar of soap (three bars of soap or three soapy water bottles required per month per household), its use would cost only US$0.15 (three packs of 30 g detergent cost US$0.09 + one plastic bottle costs US$0.06) in the first 1 month and US$0.09 for each subsequent 1 month. This cost provides a savings of US$0.90 in the first 1 month and US$0.96 for each subsequent 1 month compared with bar soap.^35^ Therefore, soapy water has the potential to be promoted as a low-cost alternative handwashing agent for Bangladeshi households who spent an average of US$0.2 per month for bar or liquid soap.^49^

Similar to other studies, our respondents preferred protecting bar soap from wastage and theft by keeping it in a safe place.^12,15^ They kept the soapy water bottles in an open place as they perceived that it was not under threat of being stolen compared with bar soap, which is consistent with findings from a school-based study in Kenya.^20^

The type of bottles used as a “dispenser” can also vary in terms of the type of top, size, presence of a holder for the bottle to sit, and storage feasibility.^17^ The dispenser tested in this study was a locally available, reclaimed used mineral water bottles manufactured by a local company, commonly available in 0.5 L, 1.5 L, and 5 L sizes.^17^ Using bottles as a dispenser in this study protected the soapy water solution from being contaminated by frequent use compared with bar soap, and functioned as a cue to action (a reminder to wash hands),^18^ especially for younger children, suggesting handwashing becomes habitual through frequent practice,^50^ with the use of a specific cue, such as an object, a person, or a message.^51,52^

Indentations on the surface of the bottles led to accumulation of dirt with frequent use. However, none of our respondents complained that they broke or lost their shape as a result of being placed outside in the sun in contrast to the experience with plastic soda bottles in Kenyan schools.^20^ Thicker, sturdier, and longer lasting commercial translucent water bottles can be resistant to the rapid degradation associated with the reclaimed bottles,^20^ though they may be prone to theft and come at greater cost. Such bottles were subsequently developed by a local manufacturer and cost approximately US$0.70–0.90/bottle. Fieldworkers punched a hole in the cap to dispense liquid from the reclaimed bottle in this study. Commercial liquid soap dispensers generally have a pump top which may minimize bottle soiling from frequent use, reducing the need to touch the bottle. However, the pump tops will increase cost, have not been designed for durability, and occasionally become blocked by un-dissolved detergent particles (F. Sultana, unpublished data). Inclusion of a pump top and a steel holder to support and keep the bottle in one place could minimize direct hand contact and thereby prevent it from becoming visibly dirty and contaminated, and being misused or misplaced. Further research can evaluate the long-term durability and willingness to pay for an added pump and a steel holder.

There are limitations of this study; primarily data were collected from only three low-income urban communities and among a small number of participants. However, the selected communities were typical of other studied Bangladeshi urban communities in terms of shared municipal water points, shared toilet facilities, common cooking areas, and handwashing stations located inside the household compounds.^9,35,39^ Although the intervention time period was relatively brief (18 months), other soapy water interventions in similar contexts over a longer duration suggest that it is feasible to consider further scale up of the soapy water system in Bangladesh.^18^

The soapy water system we evaluated was simple in design, cost-effective, replicable, popular with intervention recipient and neighboring non-recipients, and commonly shared among non-related households, suggesting a potential for scalability.^53,54^ The easy availability of the reclaimed bottle as dispensers suggests the potential for the intervention to spread to those who do not directly hear promotional messages. Therefore, this innovation has the potential to increase handwashing with soap as part of large-scale national promotions. Important areas for future research include lowering the cost of behavior change promotion potentially by using mass media, identifying systematic definitions of sustained adoption and methodology to rigorously evaluate water and sanitation interventions,^23^ and evaluating sustainability and sustained behavioral impact of such interventions.
